# Input–output analysis on the economic impact of medical care in Japan

**DOI:** 10.1007/s12199-015-0478-y

**Published:** 2015-07-21

**Authors:** Go Yamada, Yuichi Imanaka

**Affiliations:** Department of Healthcare Economics and Quality Management, School of Public Health, Graduate School of Medicine, Kyoto University, Yoshida Konoe-cho, Sakyo-ku, Kyoto-shi, Kyoto 606-8501 Japan

**Keywords:** Input–output analysis, Econometrics, Policy evaluation, Healthcare reform, Monte Carlo simulation

## Abstract

**Objectives:**

Since the Cabinet’s decision concerning the Basic Policies 2005, the Japanese government has implemented specific measures to suppress increases in national medical care expenditure. However, we believe that the economic significance of medical care should be quantified in terms of its economic impact on national medical care expenditure. No one has examined the economic impact of all medical institutions in Japan using data from a statement of profits and losses. We used an input–output analysis to quantitatively estimate economic impact of medical care and examined its estimation range with a probabilistic sensitivity analysis.

**Methods:**

To estimate the economic impact and economic impact multipliers of all medical institutions in Japan, an input–output analysis model was developed using an input–output table, statement of profits and losses, margin rates, employee income rates, consumption propensity and an equilibrium output model. Probabilistic sensitivity analysis was conducted using a Monte Carlo simulation.

**Results:**

Economic impact of medical care in all medical institutions was ¥72,107.4 billion ($661.5 billion). This impact yielded a 2.78-fold return of medical care expenditure with a 95 % confidence interval ranging from 2.74 to 2.90.

**Conclusion:**

Economic impact of medical care in Japan was two to three times the medical care expenditure (per unit). Production inducement of medical care is comparable to other industrial sectors that are highly influential toward the economy. The contribution to medical care should be evaluated more explicitly in national medical care expenditure policies.

## Introduction

Since the Cabinet’s decision concerning the Basic Policies for Economic and Fiscal Management and Structural Reform 2005 [1], the Japanese government has implemented measures to moderate healthcare costs specifically to suppress increases in national medical care expenditure. Unfortunately, these measures did not take into account the stimulatory effects of medical care on the economy. For example, to provide medical care, health sectors such as medical institutions participate in interindustry transactions as they purchase goods and services such as drugs, medical supplies, and equipment. The health sector also stimulates the economy through the provision of employment and indirectly supports the societal role of employees as consumers.

The importance of identifying the economic impact of the social security which influences the expansion of domestic demand has been emphasized by the Annual Reports on Health and Welfare in Japan [2]. National medical care expenditure supports economic activities of medical institutions and contributes to stimulation of other economic sectors involved in interindustry transactions with medical institutions. Such transactions with medical institutions comprise the so-called economic impact of medical care. The Japanese government recognize the need to clarify this impact. Thus, it is essential to quantitatively evaluate the economic impact of medical care.

Economic impact is commonly assessed by an Input–Output (I–O) analysis which uses an I–O table. Developed by Wassily Leontief, the I–O analysis is a method to systematically quantify the mutual interrelationships among the various sectors of a complex economic system [3]. The I–O table, a statistical data table for the I–O analysis, describes the flow of goods and services between all the individual sectors of a national economy over a year [4]. The I–O table for Japan is developed every 5 years by the Ministry of Internal Affairs and Communications (MIC) and 9 other ministries as a joint enterprise. Each prefecture and some cities in Japan also develop I–O tables that reflect their regional economy. The I–O table for Japan is elaborately developed and adopts a wide range of statistical reports. It is well known for its high data accuracy, reliability and accessibility. I–O analysis with this table is generally applied by government and public sectors as they evaluate the impact of a public investment, airport development or tourism industry. The economic impact estimated by the equilibrium output model using an I–O analysis evaluates the gaining economic effect on the economic front at the same time; this is separate from a direct evaluation of the policy objectives [5].

The necessity to identify the economic importance of the health sector has been widely discussed, and the I–O analysis is an established method to assess economic impact. Data from an I–O table have assessed economic impact of the health sector in such areas as cities, part of regions, counties, prefectures and a nation [6–9]. Other studies examined impact with an I–O analysis using data from a Statement of Profits and Losses (P/L), which allows for the estimation of impact on specific target regions or medical institutions [10–14]. Although each of these studies uses a different analysis model, estimated impacts range from 1.5 to 2.5 times of the final demand. However, I–O analysis and P/L data have not been used to evaluate the economic impact of the health sector, particularly the medical care of all medical institutions in Japan.

To address these concerns, the following are the objectives of this study: to use I–O analysis and P/L data to quantitatively estimate the economic impact of medical care dispensed by all medical institutions, and to examine the estimation range of the economic impact with a probabilistic sensitivity analysis.

## Materials and methods

### Data used in the analysis

We used the following data and model as basic data to develop a spreadsheet for the I–O analysis model.

### Medical revenue and medical cost

We estimated medical revenue and medical cost from the total general medical care expenditure and hospitalization meal expenses. National-level cost for the total general medical care expenditure comprised the expenditures from public expense, social insurance and personal expenses paid to medical institutions. This was equivalent to the total medical revenue for all medical institutions. Institutional-level medical cost estimated in the following section was covered by medical revenue and represents the cost of providing medical services. General medical care expenditure consisted of hospitalization expenses and hospitalization outpatient expenses of hospitals and medical clinics. These data were provided by the Estimates of National Medical Care Expenditure 2005 of the Ministry of Health, Labour and Welfare [15].

**Fig. 1:**
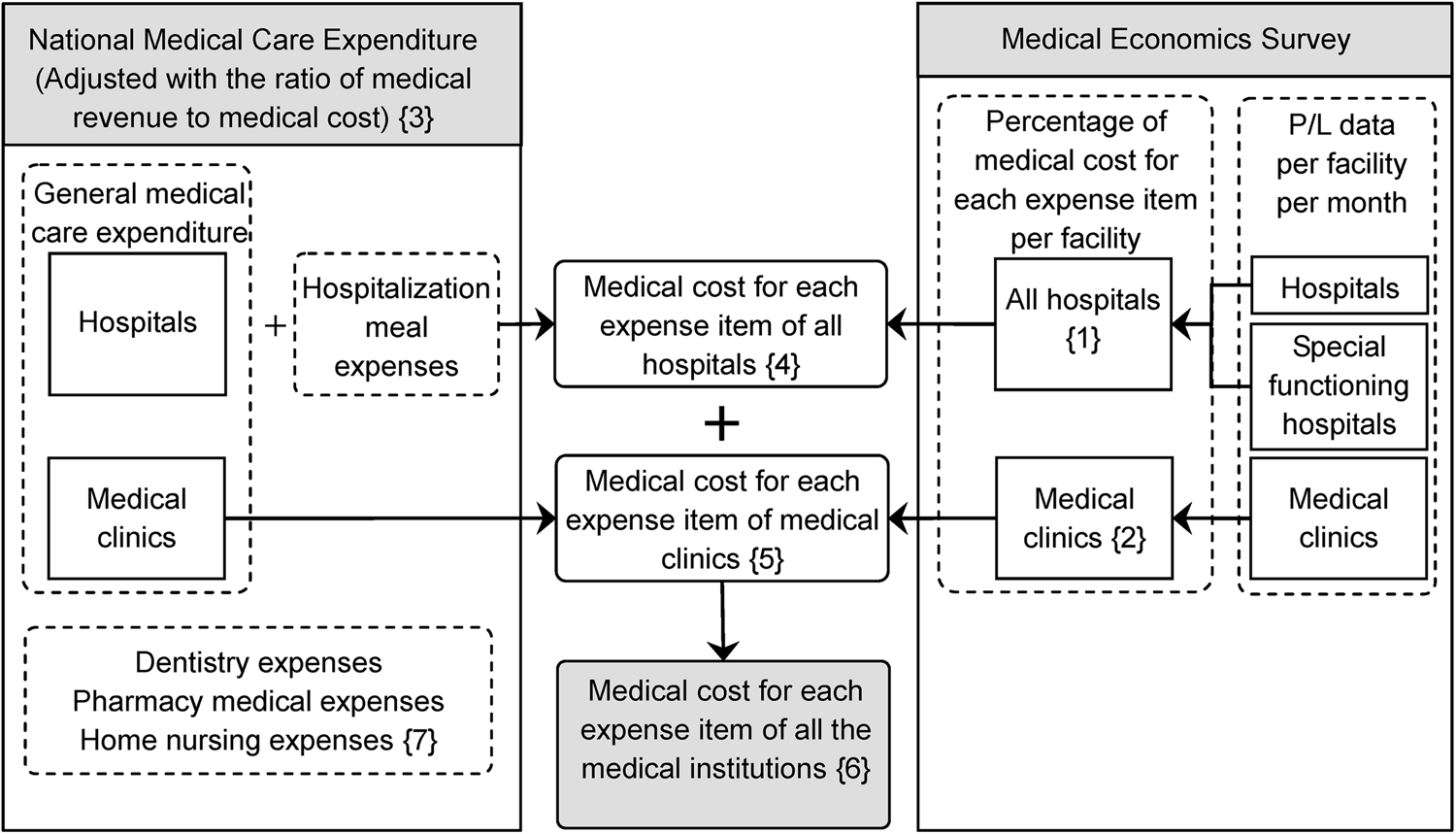
Estimation of medical cost for each expense item of all medical institutions

We also estimated medical cost from the total general medical care expenditure and hospitalization meal expenses. Since detailed expense data were not available from Estimates of National Medical Care Expenditure 2005, further calculations were required to estimate medical cost for expense items for all medical institutions. We estimated the percentage of medical cost for each expense item per facility using the P/L data per facility per month for hospitals, special functioning hospitals and medical clinics (Fig. 1). We derived the P/L data from the Medical Economics Survey, June 2005, of the Central Social Insurance Medical Council [16].

First, we estimated the percentage of medical cost for each expense item for all hospitals and special functioning hospitals. In this process, we multiplied the number of facilities with valid replies by the P/L data per facility per month for hospitals and special functioning hospitals. Next, we summed the medical cost for each expense item of hospitals and special functioning hospitals. Then, we divided this total medical cost by the total number of hospitals and special functioning hospitals. We estimated the percentage for each expense item for all the hospitals ({1} in Fig. 1). We also estimated this percentage of medical cost for each expense item for medical clinics ({2} in Fig. 1).

Compared with medical cost, medical revenue was higher for all hospitals and lower for medical clinics, according to P/L data of the Medical Economics Survey. Therefore, we estimated medical cost by multiplying medical care expenditure, which consisted of general medical care expenditure and hospitalization meal expenses, by the ratio of medical revenue to medical cost ({3} in Fig. 1).

To estimate medical cost for each expense item for all the hospitals, we allocated the total amount of general medical care expenditure and hospitalization meal expenses according to the percentage for each expense item. General medical care expenditure refers to the total amount of hospitalization expenses and hospitalization outpatient expenses of hospitals ({4} in Fig. 1). As for medical clinics, we allocated general medical care expenditure, which was a total of hospitalization expenses and hospitalization outpatient expenses of medical clinics, according to the percentage for each expense item ({5} in Fig. 1).

The sum of medical cost for each expense item for all hospitals and medical clinics estimated medical cost for each expense item for all medical institutions as basic data ({6} in Fig. 1). Of national medical care expenditure, we excluded dentistry expenses, pharmacy medical expenses and home nursing expenses for the estimation of medical revenue and medical cost ({7} in Fig. 1). Because P/L data based on the Medical Economics Survey, June 2005, of the Central Social Insurance Medical Council [16] included both nursing insurance and healthcare insurance data, excluding nursing insurance-specific data is difficult. We focused on general medical care expenditures of hospitals and medical clinics including hospitalization meal expenses covered by healthcare insurance, which amounted to 78 % of national medical care expenditure.

### I–O table

I–O analysis incorporated data from the 2000 I–O table (transactions valued at producers’ prices) 32 sectors of the MIC [17]. Trade margin rates, domestic transportation charge rates and employee income rates were calculated from this I–O table as stated in following section. The data we used, therefore, represent total economic activity in 2000. We applied 2000 I–O table 32 sectors instead of 104 sectors because medical cost used as intermediate demand was classified into 21 broad expense items and this did not correspond to 104 detailed sectors.

### Trade margin rates and domestic transportation charge rates

Trade margin rates and domestic transportation charge rates are outlined in a study of Yasuda [18] that calculated rates by dividing the margin by the purchasers’ price from the I–O table. A trade margin table and a domestic transportation charge table attached to the I–O table in some studies calculated margin rates for input to the I–O analysis model. These margin rates, however, represented all transactions in industrial sectors and were not able to cover the final demand specifically [18].

### Employee income rates

Employee income rates equaled employee income divided by domestic production from the I–O table.

### Consumption propensity

Consumption propensity was consumption expenditure divided by income of workers’ households from the 2006 Annual Report on the Family Income and Expenditure Survey, Income and Expenditure of the MIC [19]. This was one of the key data items used to estimate economic impact through consumption. As such, we evaluated the distribution of consumption propensity using a probabilistic sensitivity analysis.

### Equilibrium output model

This study used a competitive import equilibrium output model to consider exported and imported economic impact. The equilibrium output model estimated domestic induced production value (*X*) (column vector) [20]:

$$\it X = \left[ {I - \left( {I - M} \right)A} \right] ^{- 1}\left[ {\left( {I - M} \right)Y + E} \right]$$

where *I* is identity matrix (square matrix), *M* represents import coefficient (column vector), *A* is input coefficients (square matrix), *Y* is domestic final demand (column vector) and *E* is export total (column vector). Import coefficient (*M*) was the total import divided by total domestic demand of the I–O table.

### Data analysis

#### I–O analysis

Given the availability of the I–O table and applicability of the impact analysis, we used the I–O analysis for the present study. Aside from the I–O analysis, one other model that can estimate the economic impact is the allied general equilibrium analysis. This model takes advantage of a distinctive feature of the I–O table which indicates business relations of goods among industries [21]. It also corresponds to supply constraints by introducing competitive capital and labor markets, and specifically indicates an act of a household, company or government.

The I–O analysis model, or the equilibrium output model, only estimates domestic production inducement value. We allocated medical care expenditure, or medical revenue, as a direct effect in this model because economic impact was induced by production value which corresponded to an increase in medical revenue. Studies tend to use intermediate demand or medical cost, rather than final demand to estimate economic impact [10–13]. Induced production values estimated in this manner become a deducted value, as the estimation is not based on final demand but on smaller intermediate demand [22]. We therefore allocated medical care expenditure, or medical revenue, as a direct effect in the estimation of the economic impact in this study. The I–O analysis in Model 2, which allocated medical cost for direct effect, directly estimated economic impact to compare these results with previous studies that used intermediate demand.

According to prerequisites of the I–O analysis, input is proportional to output and lower costs derived from mass production, or economies of scale, are not accounted for in the analysis and vice versa [23]. The other prerequisite is that estimation of economic impact from investment value is not possible. It is difficult to identify where and when investments occur, making it unsuitable to add investment value in the I–O analysis model [24]. Hence, we excluded expense items of depreciations from P/L data from the analysis.

**Fig. 2:**
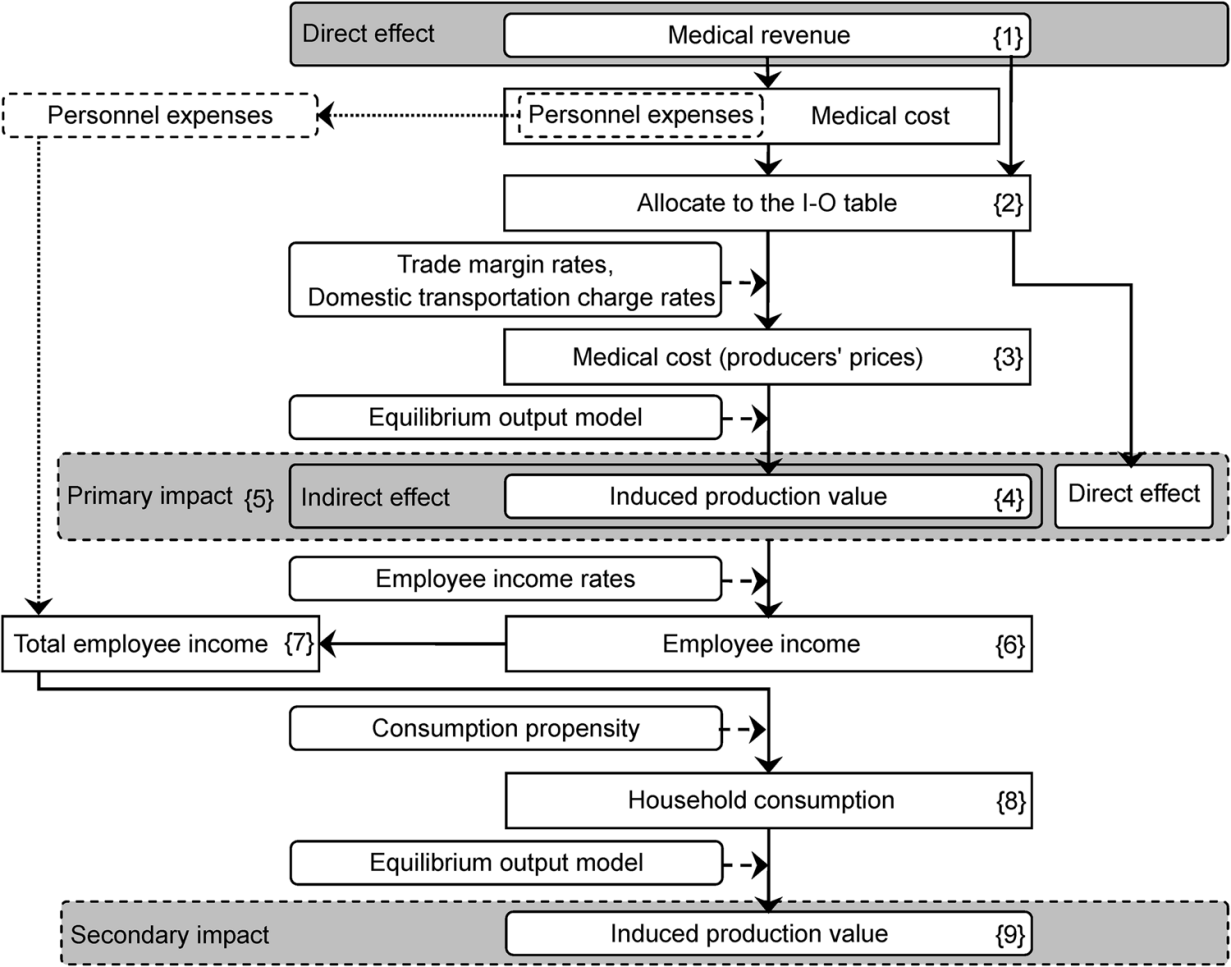
Flow chart of the I–O analysis model

The I–O analysis model consisted of two routes: a route that estimated economic impact through purchasing raw materials (raw materials' purchase route), and a route that estimated economic impact through consumption, (consumption route). The I–O analysis model of each route followed the order using the basic data (Fig. 2).

First, we analyzed the raw materials' purchase route. We allocated medical revenue as a direct effect to account for the economic impact of the purchase of raw materials ({1} in Fig. 2). We allocated medical cost to the I–O table ({2} in Fig. 2). Multiplying purchasers’ prices by trade margin rates and domestic transportation charge rates converted purchasers’ prices of medical cost, which excluded personnel expenses, to producers’ prices ({3} in Fig. 2).

Medical cost was added to the equilibrium output model as domestic final demand to estimate economic impact of the raw materials' purchase route, or indirect effect ({4} in Fig. 2). To calculate a primary impact, we added the pre-effect (direct effect) to the economic impact of the raw materials' purchase route (indirect effect) as shown in {5} in Fig. 2.

Next, we analyzed the consumption route. Employee income equaled the primary impact multiplied by employee income rates ({6} in Fig. 2). To calculate total employee income, we added personnel expenses in medical cost to employee income ({7} in Fig. 2). Multiplying consumption propensity by total employee income equaled household consumption ({8} in Fig. 2).

Household consumption was added to the equilibrium output model to estimate economic impact of the consumption route, or a secondary impact ({9} in Fig. 2). Economic impact was the sum of the primary and the secondary impact. Economic impact multipliers, or induced production coefficients, equaled this economic impact divided by direct effect.

#### Probabilistic sensitivity analysis

We used the Monte Carlo simulation for probabilistic sensitivity analysis to identify uncertainty of economic impact and economic impact multipliers, estimated using the I–O analysis, with 95 % confidence interval (CI). Monte Carlo simulations use sampling experiments to estimate the distribution of output variables that depend on several probabilistic input variables [25]. We selected consumption propensity and percentage of medical cost for each expense item from P/L data as input variables and adjusted the theoretical probabilistic distribution for these variables in order to run the simulations.

Twenty years of observed data from 1987 to 2006 collected from the Annual Report on the Family Income and Expenditure Survey, Income and Expenditure, of the MIC [19, 26] were used to calculate consumption propensity. To verify the stability of this data, we analyzed the distribution with a histogram and descriptive statistics. We fit these data to a normal probability distribution with a mean of 62 %, a standard deviation of 0.01, and a lower and upper limit range of 57 and 67 %, respectively.

For the percentage of medical cost for each expense item, we selected 19 and 13 expense items from all hospitals and medical clinics, respectively. Triangular distribution defined each of these expense items with observed data from five previous surveys conducted in September 1997, June 1999, June 2001, June 2003 and June 2005 from the Medical Economics Survey [16]. Uniform distribution defined expense items with fewer than two observed data, and triangular distribution defined the remaining expense items. The average of surveys from June 2005 and June 2003 determined the maximum likelihood value of triangular distribution. We obtained minimum and maximum values by fitting observed data in a probabilistic distribution using analysis software.

We defined indirect effect, primary impact, secondary impact, economic impact and the multipliers of each for the output variables.

We conducted repeated random sampling 1000 times according to the probability distribution of the input variables. Sampling data incorporated in the spread sheet I–O analysis model produced a normal distribution of output variables and identified the 95 % CI. Crystal Ball^®^ 2000 Professional Edition (Decisioneering, Inc.) [27] was the analysis software for the Monte Carlo simulation.

## Results

Allocation of medical revenue and medical cost of all medical institutions indicated that total medical revenue was ¥25,948.4 billion ($238.1 billion: US$1 = JPN¥109) and medical cost was ¥23,329.7 billion ($214.1 billion) (Table 1). Next to employee compensation (49 %), the largest allocation from medical cost was for chemical products (18 %).

**Table 1:** Medical revenue and medical cost for each sector of the I–O table

Expense items	Sectors of the I–O table	Value in billions
Medical revenue		¥25,948.4 ($238.1)
Inpatient revenue, outpatient revenue, other revenue	Medical service, health and social security and nursing care	¥25,948.4 ($238.1)
Medical cost		¥24,825.6 ($227.8)
Personal expenses	Compensation of employees	¥12,218.4 ($112.1)
Material costs		
Medicine	Chemical products	¥4,387.2 ($40.3)
Food material	Foods	¥196.4 ($1.8)
Medical materials and medical consumables, dental materials, others	Miscellaneous manufacturing products	¥2,025.9 ($18.6)
Expenses		
Utility costs	Electricity, gas and heat supply	¥353.4 ($3.2)
Land rents, building rents	Real estate	¥467.6 ($4.3)
Equipment rentals (medical equipment, other equipment), other utility costs	Business services	¥2,156.5 ($19.8)
Consignment costs		
Laboratory tests, dental technologies, medical clerks, others	Business services	¥1,063.2 ($9.8)
Meal service for patients	Foods	¥210.3 ($1.9)
Linen cleanings/rentals, patient gown cleanings/rentals	Personal services	¥70.0 ($0.6)
Medical waste disposals	Water supply and waste management services	¥48.7 ($0.5)
Depreciations		
Buildings, medical equipments, others	Depreciation of fixed capital	¥1,495.9 ($13.7)
Others	Business services	¥132.0 ($1.2)

The total economic impact of all medical institutions, primary and secondary impact, was ¥72,107.4 billion ($661.5 billion) with a 95 %CI ranging from ¥71,018.2 billion ($651.5 billion) to ¥75,300.0 billion ($690.8 billion) (Table 2). The impact of the raw materials' purchase route, or indirect effect, induced from the medical cost, was ¥18,474.3 billion ($169.5 billion). The impact of the consumption route, or secondary impact, stimulated from the primary impact was ¥27,684.7 billion ($254.0 billion). The economic impact multiplier (induced production coefficient), which divided the economic impact by the direct effect, was 2.78 (95 % CI 2.74–2.90).

**Table 2:** Economic impact and economic impact multipliers of all medical institutions

Impact categories	Impact value in billions	95 % CI		Economic impact multipliers	95 % CI	
Economic impact: primary impact plus secondary impact	¥72,107.4 ($661.5)	¥71,018.2 ($651.5)	¥75,300.0 ($690.8)	2.78	2.74	2.90
Primary impact	¥44,422.7 ($407.5)	¥43,214.5 ($396.5)	¥45,799.9 ($420.2)	1.71	1.67	1.77
(Direct effect: medical care expenditure, or medical revenue)	¥25,948.4 ($238.1)	–	–	1.00	–	–
(Indirect effect: economic impact of the raw materials' purchase route)	¥18,474.3 ($169.5)	¥17,266.1 ($158.4)	¥19,851.5 ($182.1)	0.71	0.67	0.77
Secondary impact: economic impact of the consumption route	¥27,684.7 ($254.0)	¥27,237.4 ($249.9)	¥30,096.2 ($276.1)	1.07	1.05	1.16

The percentage of public expenditure within national medical care expenditure is 36 %, which is equivalent to ¥12,061.0 billion ($110.7 billion) [15]. By multiplying this by the induced production value, or the economic impact, the impact of the public expenditure can be estimated, amounting to ¥25,958.7 billion ($238.1 billion). The job creation effect was estimated by multiplying the economic impact by employment rate equal to 4946 thousand employees (95 % CI 4876 thousand–5119 thousand). Employment rates were calculated by dividing the number of employees from the I–O table (employment table) [28] by the domestic production from the I–O table [17].

Economic impact estimated as Model 2 was ¥34,638.9 billion ($317.8 billion) with a 95 % CI ranging from ¥33,475.3 billion ($307.1 billion) to ¥37,227.6 billion ($341.5 billion) with an economic impact multiplier of 1.40 (95 % CI 1.38–1.46).

## Discussion

This study revealed that economic impact of all medical care by medical institutions in Japan totaled ¥72,107.4 billion ($661.5 billion), equal to 2.78 times the ¥25,948.4 billion ($238.1 billion) outlaid for medical care expenditure. Estimation of the economic impact was performed as a way to evaluate quantitatively the economic effects, including various aspects of medical care, obtained from the economic front through medical policy measures. No previous studies have identified the economic impact of medical care by all medical institutions throughout Japan using P/L data. We quantitatively identified the volume of influences that medical care expenditure has on the Japanese economy using P/L data. To date, no probabilistic sensitivity analysis has been conducted on the economic impact. We used the Monte Carlo simulation for the probabilistic sensitivity analysis and indicated the uncertainty derived from the consumption propensity and P/L data in the I–O analysis model with an estimation range of the economic impact.

### Contributions to the direct effect

**Fig. 3:**
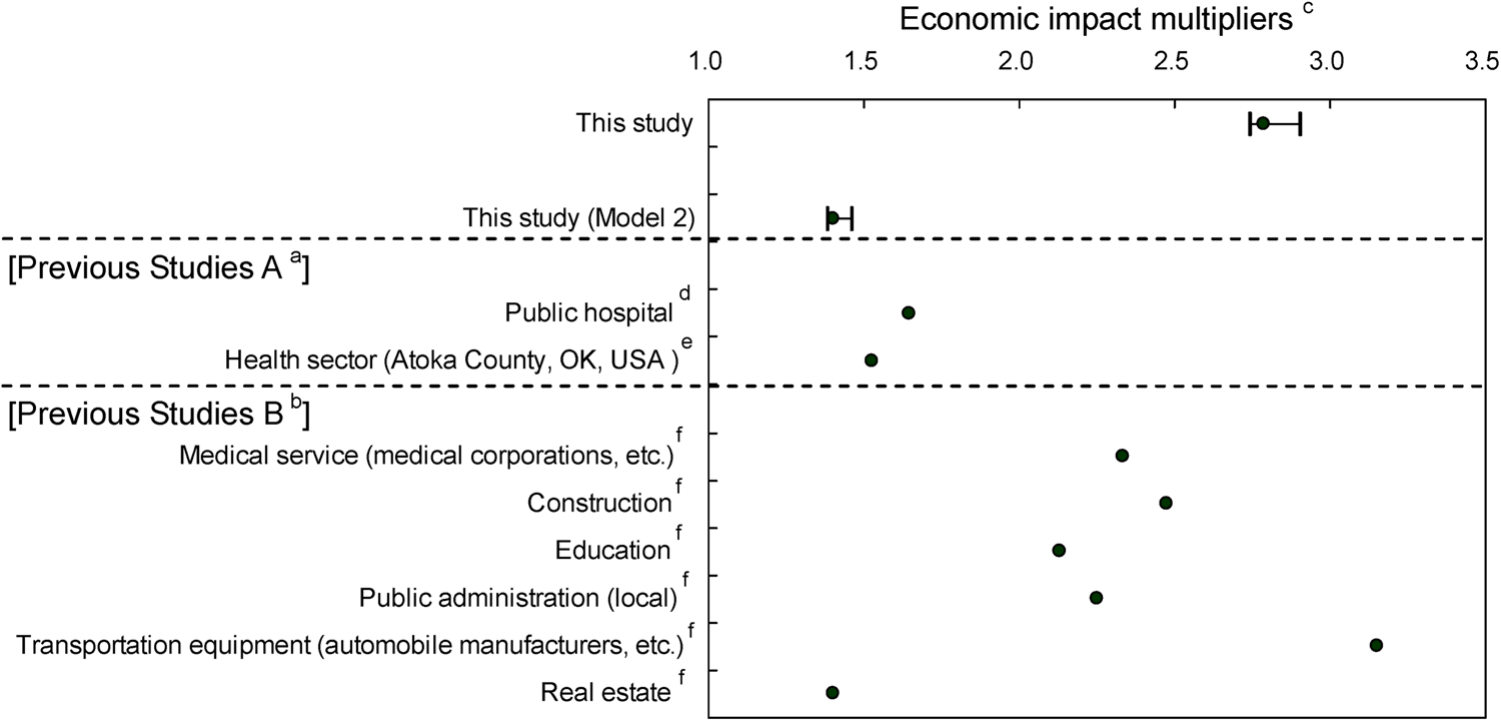
Comparison of economic impact multipliers from previous studies. ^a^Previous studies which estimated economic impact multipliers using P/L data and the I–O table in the same manner as our study. In contrast to the analysis model from this study, these studies applied medical cost instead of medical care expenditure, or medical revenue. ^b^Previous studies that analyzed data from the I–O table to estimate economic impact multipliers. ^c^Economic impact multipliers of this study as well as Model 2 indicate point estimates and 95 % CI. ^d^Doi and Nakano [12]. ^e^Doeksen and Schott [10]. ^f^Tsukahara [5]

Estimates of economic impact in all medical institutions were higher in this study compared to the group of Previous Studies A outlined in Fig. 3. Among those studies, Doi and Nakano [13] estimated economic impact of a public hospital using P/L data in the same manner as our study to be 1.64 times medical care expenditure. The disparities between this study and the Previous Studies A resulted from differences in accounting for contributions to the direct effect. Our study applied medical care expenditure, or medical revenue, to the direct impact, whereas Doi and Nakano [13] applied medical cost. In consideration of their analysis model, we examined economic impact multipliers, or induced production coefficients, from Model 2 and applied medical cost to the direct effect. As a result of applying this method, the Model 2 multiplier was 1.40, which was similar to results from their study. The economic impact of their study deducted the amount of medical revenue, resulting in a relatively small economic impact.

A direct comparison of results from them with results from our study is difficult because they estimated the economic impact based on one prefecture. In addition, other than medical cost, they included nonmedical cost not included in this study, such as store cost and visitor travel cost. Most importantly, economic impact of all medical institutions in this study was estimated with a theoretically valid methodology compared to the results of Doi and Nakano [13], which estimated economic impact excluding medical care expenditure, or medical revenue, which must be applied to direct effect.

### Economic impact multiplier

Tsukahara [6] of Previous Studies B analyzed data from the I–O table to estimate economic impact multipliers instead of using P/L data. This study identified the medical service sector economic impact multiplier to be 2.33 and transportation equipment sector, including automobile manufacturers, to be 3.15, if consumption propensity equaled 60 %. In comparison with these medical service sector results, medical care of all medical institutions from our study evidently indicated an elevated economic impact multiplier of 2.78. Given these differences in analysis models, further studies are needed to explore the effects of analytical differences on results.

### Uncertainty of consumption propensity

Tsukahara [6] noted that rather than a reliance on a flat consumption propensity, the adoption of a marginal propensity to consume, a percentage of consumption that alters when income increases, was preferable. In response to this uncertainty, we incorporated a normal distribution into the probabilistic sensitivity analysis with a consumption propensity ranging from 57 to 67 %.

### Study limitations

This study estimated economic impact of the medical care of all medical institutions from medical care expenditure, a total of general medical care expenditure and hospitalization meal expenses out of national medical care expenditure. However, we excluded dentistry expenses, pharmacy medical expenses and home nursing expenses in this study because of unavailability of detailed data. These expenses accounted for 22 % of national medical care expenditure; therefore, further analysis incorporating these excluded data is needed to identify the true economic impact of all medical care.

## Conclusion

In conclusion, medical care expenditures represent a resource for medical institutions that allow them to continuously provide medical services which contribute to the well-being of the whole nation. As such, any shortage in this expenditure can severely affect the quantity and quality of medical services for those who need medical care. It also supports the economic activities of medical institutions and helps stimulate other economic sectors involved in interindustry transactions with medical institutions. These transactions, in turn, stimulate the economy through the provision of employment and indirectly support the societal role of employees as consumers.

This study identified the economic impact of medical care other than the job creation effect in Japan to be 2–3 times the input (in units) allocated from medical care expenditure. Medical care had as strong a production inducement as other sectors recognized as industries with high economic effects. The Japanese government had taken measures to suppress national medical care expenditure; however, when evaluating national medical care expenditure policies, it is necessary to evaluate the contribution to medical care more explicitly, particularly regarding the economic stimulation through the maintenance and production of employment in various industries related to medical care.

Besides the economic impact, evaluation of the medical institutions should consider the impact of medical care on society as well. Further studies are needed to quantify the impact and importance of medical institutions in terms of benefit toward people who live in the society.

## References

[1] Cabinet Office. Basic Policies for Economic and Fiscal Management and Structural Reform 2005. http://www.kantei.go.jp/jp/singi/keizai/kakugi/050621honebuto.pdf. **(in Japanese)** 11 Feb 2013.

[2] Ministry of Health, Labour and Welfare. Annual Reports on Health and Welfare 1999. http://www.mhlw.go.jp/english/wp/wp-hw/index.html 11 Feb 2013.

[3] Leontief W (1986). Input–output economics.

[4] Leontief W (1966). Input–output economics.

[5] Yasuda H (2001). Time of creation and policy development. Finance Region.

[6] Tsukahara Y (1996). Input–output analysis of medical activity: the interaction model of goods industries and service industries. Jpn J Health Econ Policy.

[7] Miyazawa K (2000). Input–output analysis and economy of health care in the aging welfare society. Jpn J Health Econ Policy.

[8] Matsuda S, Murata H, Funatani F (1997). Input–output analysis of investment in health sector of Kitakyushu city. Jpn J Health Econ Policy.

[9] Lichty RW, Jesswein WA, McMillan DJ (1986). Estimating medical industry impacts on a regional economy. Med Care.

[10] Doeksen GA, Johnson T, Willoughby C. Measuring the economic importance of the health sector on a local economy: a brief literature review and procedures to measure local impacts: Southern Rural Development Center, 1997.

[11] Doeksen GA, Schott V. Economic importance of the health-care sector in a rural economy. Rural Remote Health 2003;3:1–6. 15877487

[12] Scorsone EA, Garcia S, Adams B (2001). Economic impact of Knox County Hospital.

[13] Doi E, Nakano C (2001). The input–output analysis about the effect of public hospital on regional economy. Econ Rev Shizuoka Univ.

[14] House DR, Fry CL, Brown LJ (2004). The economic impact of dentistry. J Am Dental Assoc.

[15] Ministry of Health, Labour and Welfare. Estimates of National Medical Care Expenditure 2005. Tokyo, Health and Welfare Statistics Association, 2008. **(in Japanese)**.

[16] Central Social Insurance Medical Council. Medical economics survey 1997–2005. **(in Japanese)**.

[17] Ministry of Internal Affairs and Communications. 2000 Input-Output Tables for Japan. 2004. http://www.stat.go.jp/english/data/io/io00.htm 11 Feb 2013.

[18] Yasuda H (2005). Input–output analysis practice (1). Input–Output Anal Innov I–O Tech.

[19] Ministry of Internal Affairs and Communications. Annual report on the family income and expenditure survey 2000–2006, income and expenditure. http://www.e-stat.go.jp/SG1/estat/Xlsdl.do?sinfid=00000033273C. **(in Japanese)** 11 Feb 2013.

[20] Ministry of Internal Affairs and Communications (2005). 2000 Input output tables for Japan.

[21] Ministry of Internal Affairs and Communications. Policy evaluation report on screening system, 2004. http://www.soumu.go.jp/menu_news/s-news/daijinkanbou/040402_3_a.pdf. **(in Japanese)** 11 Feb 2013.

[22] Yasuda H (2005). Input–output analysis practice (2). Input–Output Anal Innov I–O Tech.

[23] Murata Y, Ito K, Koshikuni M (2005). Introduction to economic forecasting with PC.

[24] Yasuda H (2005). Input–output analysis practice (3). Input–Output Anal Innov I–O Tech.

[25] Evans JR, Olson DL (1998). Introduction to simulation and risk analysis.

[26] Ministry of Internal Affairs and Communications. Annual report on the family income and expenditure survey 1963–2007, income and expenditure. http://www.stat.go.jp/data/kakei/longtime/zuhyou/a18-2.xls. **(in Japanese)** 11 Feb 2013.

[27] Crystal Ball^®^ 2000 Professional Edition [computer program] Version 5.2 (Denver, Decisioneering, Inc.).

[28] Ministry of Internal Affairs and Communications. 2000 I–O table (employment table). Research Institute of Economy, Trade and industry. **(in Japanese)**.

